# PARAFAC-Assisted Excitation–Emission Matrix Fluorescence for Minimal-Preparation Co-Determination of Norfloxacin and Metoclopramide in Human Urine: Overcoming Severe Concentration Imbalance Without Chromatography

**DOI:** 10.3390/molecules31213984

**Published:** 2026-11-04

**Authors:** Mahmoud M. Elkhoudary, Randa A. Abdel Salam, Mohamed S. Abdel-Maksoud, Ghada M. Hadad

**Affiliations:** 1 Department of Pharmaceutical Chemistry, Faculty of Pharmacy, Horus University-Egypt, New Damietta 34518, Egypt; 2 Department of Pharmaceutical Analytical Chemistry, Faculty of Pharmacy, East Port Said National University, Port Said 42526, Egypt; 3 Department of Pharmaceutical Analytical Chemistry, Faculty of Pharmacy, Suez Canal University, Ismailia 41522, Egypt; 4 Department of Pharmacology and Toxicology, Faculty of Pharmacy, University of Tabuk, Tabuk 71491, Saudi Arabia

**Keywords:** excitation–emission matrix (EEM), fluorescence, green bioanalysis, metoclopramide, norfloxacin, PARAFAC, second-order advantage, urinary excretion profiling

## Abstract

Direct simultaneous determination of norfloxacin (NOR) and metoclopramide (MET) in human urine is severely complicated by intense biological autofluorescence, spectral overlap, and an extreme concentration disparity arising from clinical co-administration (400 mg NOR vs. 10 mg MET; dose ratio 40:1). Multiway chemometrics offers a chromatography-free approach to resolving fluorophores, but biological background and extreme concentration disparities can complicate global PARAFAC modeling. Herein, we demonstrate that this limitation can be completely overcome using a dual-submatrix excitation–emission matrix (EEM) PARAFAC strategy in human urine after a simple 10-fold dilution with pH 3.0 aqueous buffer, omitting extraction, derivatization, and organic solvents. By partitioning the measured EEM array into two dedicated sub-arrays (**X**_NOR_: λ_ex_ 260–310 nm, λ_em_ 440–500 nm; **X**_MET_: λ_ex_ 260–310 nm, λ_em_ 380–410 nm), rank deficiency is circumvented, rotational ambiguity is eliminated, and trilinearity is fully preserved. External calibration in spiked urine yielded linear dynamic ranges of 1000–6000 ng mL^−1^ for NOR (*R*^2^ = 0.9981) and 15–80 ng mL^−1^ for MET (*R*^2^ = 0.9995). Validation across independent test mixtures showed excellent mean recoveries of 100.79 ± 4.57% for NOR and 101.86 ± 4.06% for MET, with root mean square errors of prediction (RMSEP) of 144.65 and 2.38 ng mL^−1^, respectively. Net analyte signal figures of merit demonstrated high sensitivity and limits of detection of 119.87 ng mL^−1^ (NOR) and 3.16 ng mL^−1^ (MET). The validated method was successfully applied to urinary excretion monitoring in five healthy volunteers following oral co-administration of commercial tablets (Noracin^®^ 400 mg and Primperan^®^ 10 mg), recovering 24.89 ± 4.63% (NOR) and 13.59 ± 9.27% (MET) of the administered dose over 72 h. Multi-metric greenness and practicality assessments (AGREE: 0.68, ComplexMoGAPI: 69, CaFRI: 63.1, BAGI: 75.0) confirmed substantial sustainability advantages over conventional chromatographic workflows. This study revitalizes PARAFAC as an ultra-green, chromatography-free tool for modern pharmaceutical bioanalysis.

## 1. Introduction

Simultaneous determination of co-administered therapeutic agents in human biological fluids remains a cornerstone of clinical pharmacokinetics, therapeutic drug monitoring, and bioequivalence evaluation. Antibacterial and antiemetic/prokinetic agents may be co-administered in foodborne bacterial gastroenteritis, which remains a major global health burden [1]: fluoroquinolones have been used to treat and eradicate Salmonella infection [2,3,4], whereas gastrointestinal infection can be followed by persistent dyspeptic symptoms [5] that are managed with antidopaminergic antiemetic/prokinetic agents such as metoclopramide [6,7]. Specifically, norfloxacin (NOR; 1-ethyl-6-fluoro-4-oxo-7-(piperazin-1-yl)-1,4-dihydroquinoline-3-carboxylic acid) is a broad-spectrum synthetic fluoroquinolone antibacterial targeting bacterial DNA gyrase and topoisomerase IV, exhibiting high urinary excretion (~30% unchanged drug) and native fluorescence [8,9,10,11,12]; fluoroquinolones, including NOR, are also targeted by multiclass LC–MS/MS residue methods in animal tissue and wastewater [13,14]. Metoclopramide (MET; 4-amino-5-chloro-N-(2-(diethylamino)ethyl)-2-methoxybenzamide) is a dopamine D2-receptor antagonist and 5-HT4 agonist used as an antiemetic and prokinetic agent to alleviate nausea and accelerate gastric motility [6,7]; it exhibits native fluorescence, and spectrophotometric, spectrofluorimetric, and chromatographic methods have been reported for its determination [15,16,17,18,19,20,21,22].

Despite their frequent clinical co-administration, simultaneous quantification of NOR and MET in human urine presents a severe analytical hurdle. In clinical dosage regimens, NOR is administered in high therapeutic doses (typically 400 mg per unit), whereas MET is administered at comparatively minor doses (typically 10 mg per unit). This 40-fold dosage disparity results in an extreme urinary concentration imbalance (often exceeding a 75:1 molar/mass ratio in excreted urine), while the biological matrix itself exhibits formidable, heterogeneous autofluorescence arising from endogenous fluorophores such as pteridines and pyridoxic acid [23].

To date, most analytical methods reported for NOR and MET in biological fluids and pharmaceutical matrices have relied on high-performance liquid chromatography (HPLC) with UV or fluorescence detection [11,12,19,20] or liquid chromatography–tandem mass spectrometry (LC–MS/MS) [21,22], whereas the reported spectrophotometric and spectrofluorimetric methods address each drug separately rather than their simultaneous determination [9,10,15,16,17,18]. While chromatographic techniques remain the gold standard for separating multi-component mixtures, they impose heavy operational penalties: lengthy chromatographic run times (10–30 min per sample), high instrument capital and maintenance costs ($250,000–$500,000), column fouling by biological macromolecules, and substantial consumption of toxic organic solvents (acetonitrile, methanol) alongside complex sample preparation (solid-phase extraction, liquid–liquid extraction, or protein precipitation). In an era governed by Green Analytical Chemistry (GAC) [24,25,26,27] and White Analytical Chemistry (WAC) [28] paradigms, the reflex reliance on chromatography for high-throughput urinary screening represents an unsustainable ecological and economic bottleneck.

In contrast, spectrofluorimetry offers unmatched speed, operational simplicity, ambient measurement conditions, and exceptional native sensitivity for conjugated fluorophores. However, conventional zero-order and first-order fluorescence methods cannot resolve severe spectral overlap or uncalibrated matrix background without prior separation. To unlock the power of optical spectroscopy, multiway chemometric calibration of second-order data, particularly excitation–emission matrix (EEM) fluorescence, has emerged as one of the most promising analytical frontiers [29,30,31,32,33,34,35,36,37,38,39,40,41,42,43,44]. Parallel factor analysis (PARAFAC), pioneered in chemistry by Bro [33] with subsequent second-order calibration applications reported by several research groups [29,30,31,32,36,37,38,39,40,41,42,43,44], achieves the celebrated ‘second-order advantage’ (the mathematical deconvolution of target analyte profiles in the presence of unmodeled, uncalibrated interferents). This capability effectively constitutes ‘mathematical chromatography’—resolving individual chemical components within a 1-cm cuvette in 60 s without physical separation columns.

Despite this potential, chromatographic platforms remain the routine choice in pharmaceutical bioanalysis, whereas much recent EEM-PARAFAC research has focused on environmental dissolved organic matter (DOM), including DOM–metal binding [45]. Rank deficiency, noise, and interferences in excitation–emission data also continue to require dedicated chemometric treatment [46]. A relevant analytical challenge is that classical global PARAFAC models can struggle when applied to real-world drug co-administration regimens characterized by extreme concentration disparities. When one analyte is present at overwhelming concentrations relative to another as in the 40:1 NOR/MET pair, the dominant fluorophore together with endogenous urine background induces severe signal masking, rank deficiency, inner-filter effects, and loss of mathematical trilinearity, causing standard global PARAFAC models to collapse.

Herein, we demonstrate that this limitation can be resolved through an intelligent dual-submatrix spectral partitioning strategy. By establishing two dedicated PARAFAC models spanning tailored excitation–emission sub-domains (**X**_NOR_ and **X**_MET_), we restore trilinearity, eliminate rotational ambiguity, and achieve accurate simultaneous co-determination of NOR and MET directly in human urine after a simple 10-fold dilution with aqueous buffer, completely omitting organic extraction, derivatization, and chromatography. The method was validated with reference to the ICH M10 bioanalytical principles [47,48], successfully applied to urinary excretion monitoring in healthy human volunteers, and systematically benchmarked using state-of-the-art sustainability metrics (AGREE, ComplexMoGAPI, CaFRI, BAGI) [25,26,27,49]. This work demonstrates that multiway mathematical chromatography can offer a greener, solvent-free alternative to chromatographic workflows for this bioanalytical application.

## 2. Results and Discussion

### 2.1. Spectroscopic Properties and Parameter Optimization

**Figure 1:**
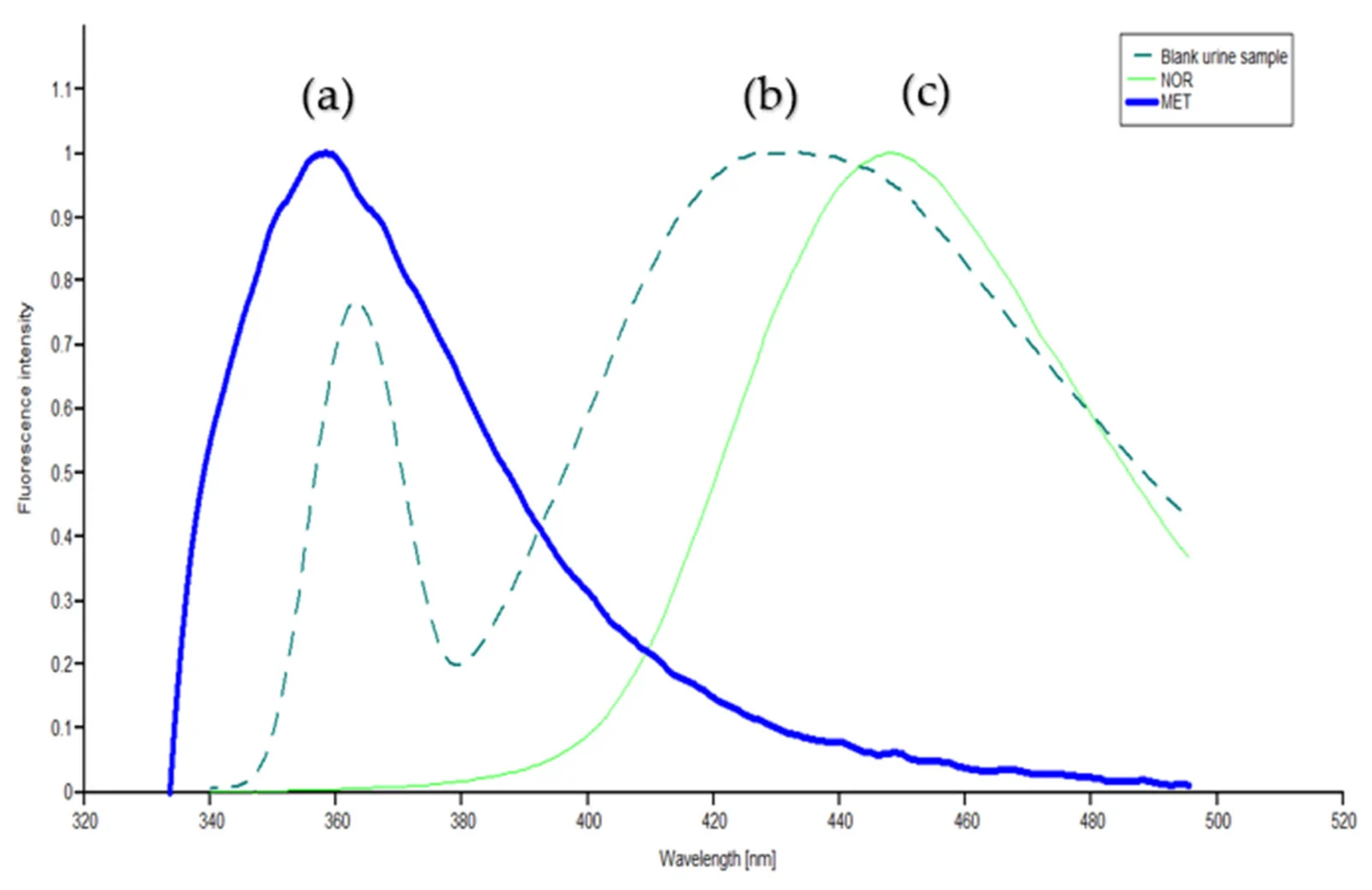
Overlay normalized emission spectra of (a) MET, (b) blank urine and (c) NOR at excitation wavelength 310 nm.

**Figure 2:**
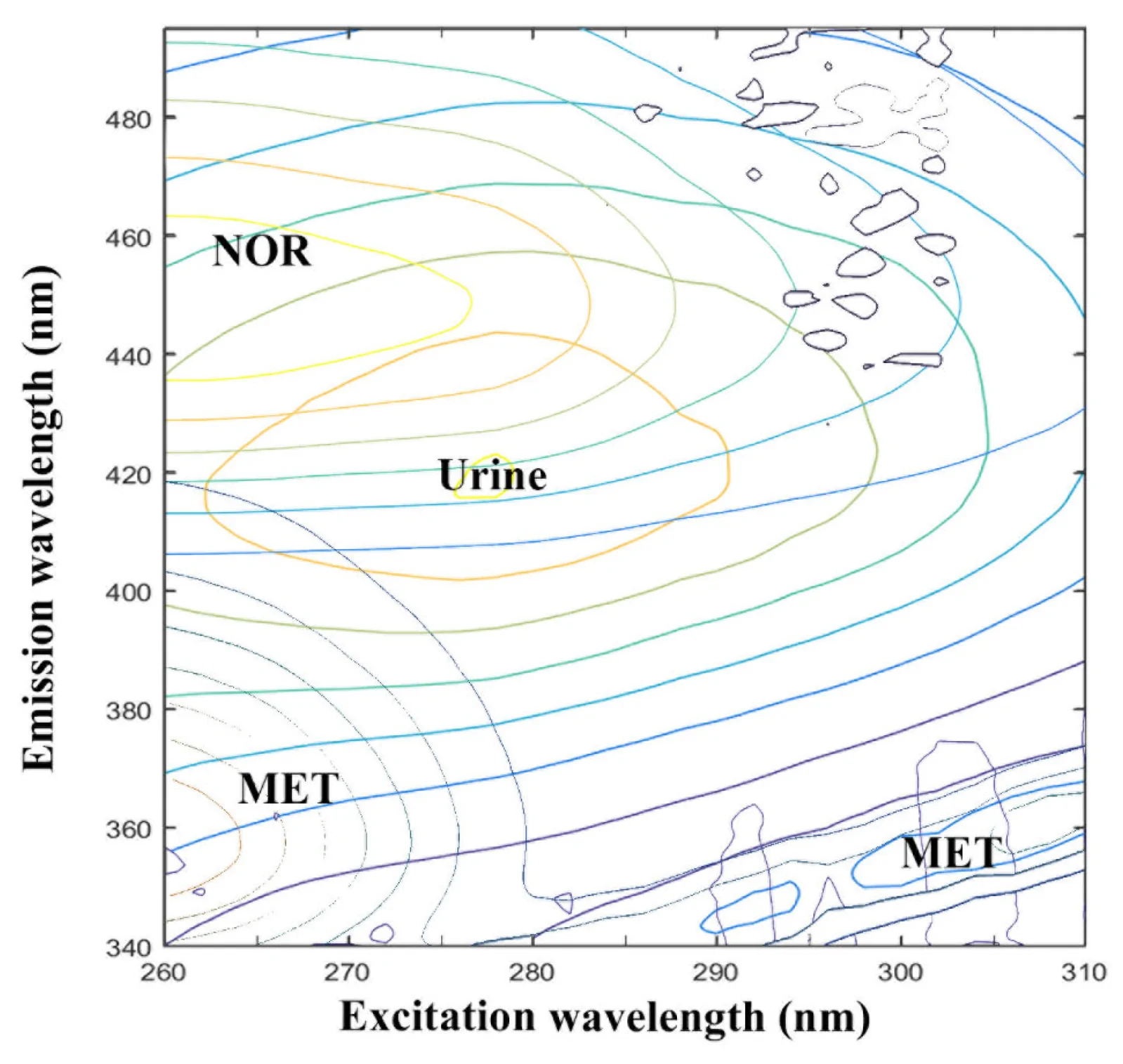
Overlaid excitation–emission matrix (EEM) fluorescence contour plots for aqueous solutions of metoclopramide (MET, 40 ng mL^−1^; dark blue/purple contours), norfloxacin (NOR, 3000 ng mL^−1^; light blue/cyan contours), and a blank urine sample (yellow/orange contours). Lines represent regions of equal fluorescence intensity across the scanned excitation (260–310 nm) and emission (340–500 nm) wavelengths.

**Figure 3:**
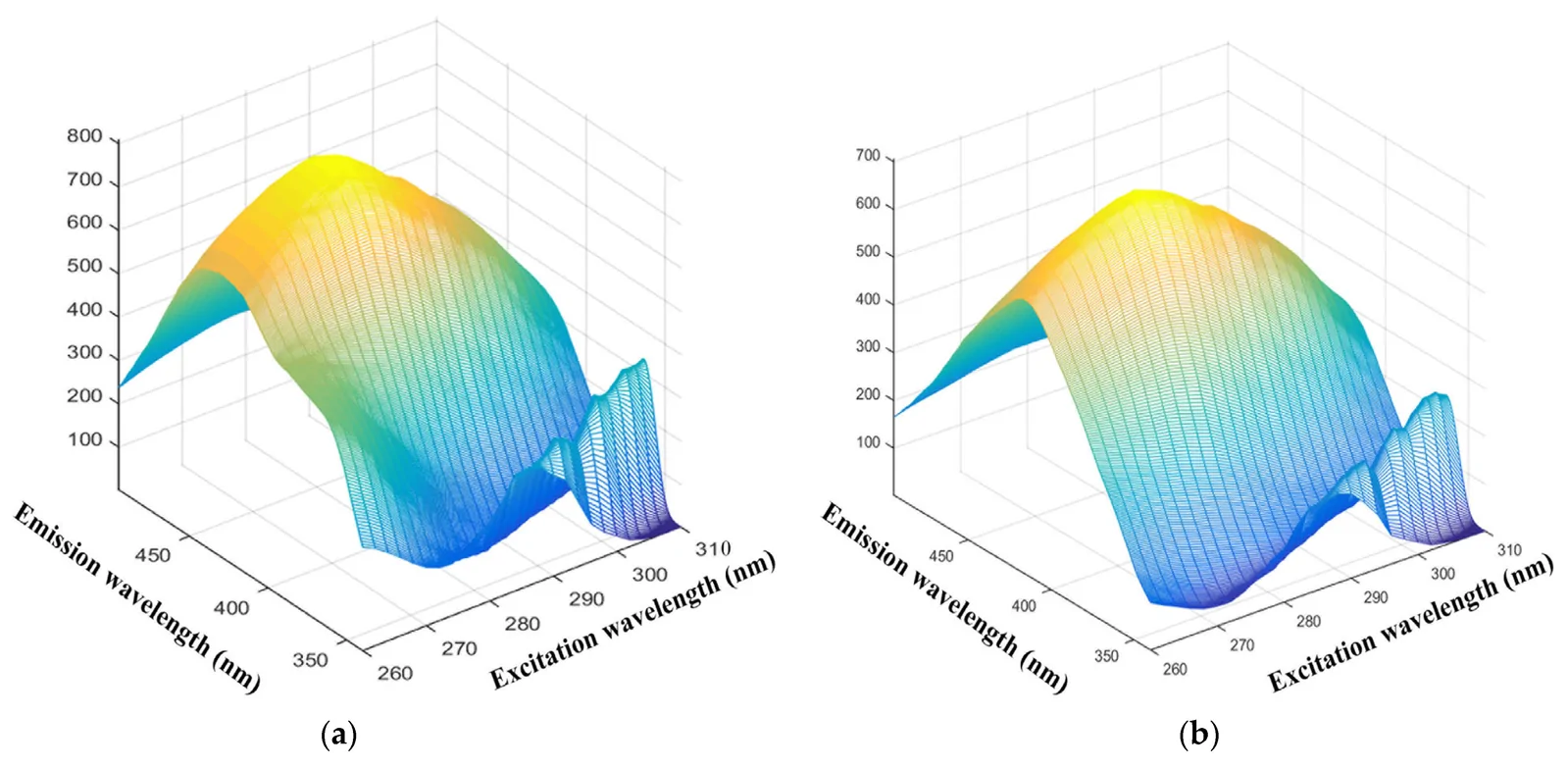
Excitation and emission matrix of (**a**) a calibration spiked sample of 40 ng mL^−1^ metoclopramide (MET) and 2250 ng mL^−1^ norfloxacin (NOR) and (**b**) blank urine sample.

Preliminary spectroscopic investigation revealed that MET exhibits an excitation maximum at 310 nm and an emission maximum at 360 nm, whereas NOR displays excitation maxima at 280 and 320 nm with broad emission centered at 450 nm (Figure 1). When diluted in blank human urine, endogenous biological fluorophores contribute substantial, broad autofluorescence spanning λ_ex_ 260–340 nm and λ_em_ 380–520 nm (Figure 1, Figure 2 and Figure 3). The influence of medium pH was evaluated over the range pH 1.0–9.0 using various buffer systems. Because MET is present in urine at levels 40–75 times lower than NOR, optimizing conditions for MET signal generation was of paramount importance. MET emission intensity peaked sharply at pH 3.0 in 0.1 M phosphate buffer; at pH < 2.0, fluorescence was quenched by excessive acid protonation, whereas at pH > 4.5, neutral species formation led to nearly complete signal extinction. NOR maintained robust, stable fluorescence at pH 3.0. The addition of organic modifiers (methanol, ethanol, isopropanol, acetonitrile) was evaluated to assess whether fluorescence could be further enhanced. However, organic solvents decreased the emission intensity of MET while dramatically increasing baseline noise. Consequently, pure aqueous 0.1 M phosphate buffer (pH 3.0) was selected as the measurement medium, perfectly aligning with green analytical principles by eliminating hazardous solvents.

### 2.2. Spectral Overlap, Matrix Interference, and Dual-Submatrix PARAFAC Resolution

Overlay contour plots (Figure 2) and 3D EEM mesh profiles (Figure 3) illustrate the profound analytical challenge: NOR emission overlaps extensively with urine autofluorescence, while the trailing edge of MET emission is engulfed by the rising flank of NOR. When combined with the massive concentration imbalance (NOR: 1000–6000 ng mL^−1^ vs. MET: 15–80 ng mL^−1^), direct univariate or first-order derivative spectrofluorimetry failed completely. Initial attempts to build a global three-way PARAFAC model across the entire acquired domain (λ_ex_ 260–310 nm, λ_em_ 340–500 nm) proved unsuccessful: due to the overwhelming photon count from NOR, the alternating least squares algorithm converged onto two components corresponding solely to NOR and urine background, completely failing to isolate MET. Truncating the dataset to emphasize MET suppressed NOR recovery. This behavior illustrates the limitations of the global model for the concentration imbalance and matrix examined in this study.

**Figure 4:**
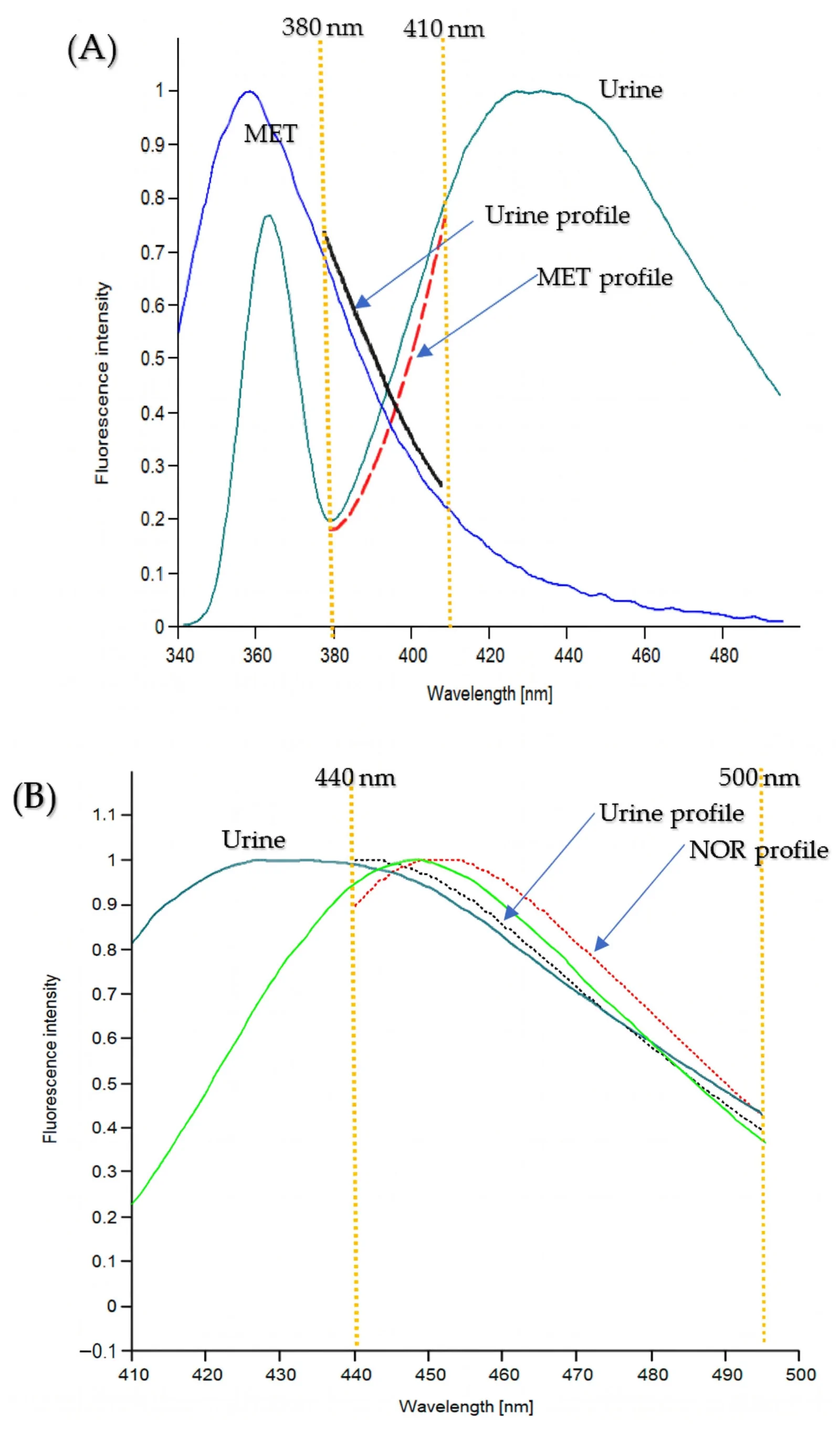
Normalized emission profiles which were resolved from (**A**) **X**_MET_ and (**B**) XNOR arrays of spiked calibration urine samples using external calibration strategy, Solid curves show the full normalized emission profiles of MET (blue), NOR (green), and urine (teal). Vertical dotted yellow lines delineate the selected resolution windows: 380–410 nm for MET and 440–500 nm for NOR. Inset profiles within these boundaries display the modeled analyte response (red dashed/dotted curves) alongside the urine background decay (black lines).

To overcome this bottleneck, we implemented a dual-submatrix spectral partitioning strategy, dividing the acquired EEM dataset into two statistically optimized, non-overlapping sub-arrays: (1) Sub-array **X**_MET_, tailored for metoclopramide quantification, spanning λ_ex_ 260–310 nm (26 wavelengths, 2 nm step) and λ_em_ 380–410 nm (61 wavelengths, 0.5 nm step), resulting in a data tensor of dimensions 16 × 61 × 26. In this sub-window, MET emission is maximized while the bulk of NOR emission is excluded. PARAFAC decomposition with *F* = 3 components (MET, residual NOR tail, and background urine) achieved optimal deconvolution (CORCONDIA = 94.2%, Fit = 99.85%), isolating the authentic MET emission profile (Figure 4A). As detailed in the factor diagnostic analysis in Table S1, increasing components to *F* = 4 triggered model overparameterization and a precipitous collapse of core consistency to 38.6%. (2) Sub-array **X**_NOR_, tailored for norfloxacin quantification, spanning λ_ex_ 260–310 nm (26 wavelengths) and λ_em_ 440–500 nm (111 wavelengths), yielding an array of dimensions 16 × 111 × 26. In this spectral window, MET emission is completely absent, allowing a 2-component PARAFAC model (*F* = 2: NOR and urine background) to extract NOR sample loadings with outstanding fidelity (CORCONDIA = 98.7%, Fit = 99.92%, Figure 4B; Table S1). Scattering artifacts (Rayleigh and Raman) were eliminated through aqueous buffer blank subtraction combined with targeted spectral window boundary selection, avoiding the need for mathematical interpolation. This dual-submatrix architecture definitively resolves the rank deficiency problem, restoring the second-order advantage for extreme concentration ratios. Specifically, the dual-submatrix design isolates the quantification windows geometrically from severe scattering regimes: for **X**_NOR_ (λ_ex_ 260–310 nm, λ_em_ 440–500 nm), 1st-order Rayleigh scatter (≤310 nm) and the solvent water Raman scattering band (285–348 nm) terminate at least 92 nm below the 440 nm emission boundary, while 2nd-order Rayleigh scatter (2λ_ex_ = 520–620 nm) lies safely above 500 nm. For **X**_MET_ (λ_ex_ 260–310 nm, λ_em_ 380–410 nm), 1st-order Rayleigh terminates 70 nm below the window, and water Raman scattering (peak at ~346 nm for λ_ex_ 310 nm) is separated by 34 nm from the lower emission threshold. Furthermore, the 10-fold dilution with 0.1 M phosphate buffer (pH 3.0) normalizes refractive index, viscosity, and background turbidity across individual urine samples (>90% uniform aqueous buffer matrix). Testing across six distinct donor urine blanks confirmed that residual inter-individual scattering differences are readily accommodated by PARAFAC’s second-order advantage into the unmodeled background factor without producing false-positive signals or biasing analyte loadings (found *a_if_* < LOD in all unspiked donor blanks). To rigorously benchmark the dual-submatrix strategy, a direct quantitative and diagnostic comparison was performed against traditional global PARAFAC and the Second-Order Standard Addition Method (SOSAM), with comprehensive metrics compiled in Table S2. When traditional global PARAFAC was applied across the entire EEM domain (16 × 121 × 26), the 40–75-fold concentration disparity caused the massive photon count of NOR and urinary autofluorescence to dominate the alternating least squares (ALS) sum-of-squares objective function. Consequently, MET variance was treated as residual noise, triggering severe factor degeneracy (CORCONDIA plummeted to 54.3%) and catastrophic MET prediction errors (RMSEP 28.65 ng mL^−1^, %RE up to 48.5%). Conversely, while SOSAM achieved satisfactory accuracy (%RE ≤ 5.10%) by establishing sample-specific standard additions, it requires at least 3 standard additions per unknown sample. For our 72-h clinical urinary excretion protocol (5 volunteers × 12 timepoints = 60 samples), SOSAM would demand 240–300 separate EEM acquisitions and excessive manual pipetting, rendering longitudinal monitoring logistically prohibitive. The proposed dual-submatrix PARAFAC delivers SOSAM-equivalent quantitative accuracy (MET RMSEP 2.38 ng mL^−1^, %RE ≤ 6.76%) using a single unified calibration set in only ~1.0 min per sample.

### 2.3. Calibration Performance, Validation, and Figures of Merit

Quantitative calibration was performed by regressing PARAFAC sample-mode loadings (*a_if_*) against nominal analyte concentrations across 10 calibration spiked urine mixtures (Table 1). As detailed in Table 2, both models exhibited outstanding linearity: *R*^2^ = 0.9981 for NOR (1000–6000 ng mL^−1^) and *R*^2^ = 0.9995 for MET (15–80 ng mL^−1^). The corresponding linear regression equations, slopes, intercepts, and standard errors are compiled in Table S3. Root mean square errors of calibration (RMSEC) were 70.77 ng mL^−1^ (NOR) and 0.86 ng mL^−1^ (MET), with mean calibration recoveries of 99.81 ± 1.83% and 99.95 ± 2.58%, respectively. The predictive performance was independently audited using 5 external validation mixtures prepared in biological urine (Table 3). Mean validation recoveries were 100.79 ± 4.57% for NOR and 101.86 ± 4.06% for MET, yielding root mean square errors of prediction (RMSEP) of 144.65 ng mL^−1^ (NOR) and 2.38 ng mL^−1^ (MET). Comprehensive chemometric modeling parameters and algorithmic diagnostics are summarized in Table 4, confirming that both models satisfied strict trilinearity and convergence criteria. Multivariate figures of merit (Table 5) confirmed excellent analytical capability: sensitivity (SEN) was 1.12 for NOR and 1.96 for MET; selectivity (SEL) was 0.42 for NOR and 0.15 for MET. The lower SEL for MET accurately captures its challenging position nestled within the urine/NOR fluorescence background, yet its high analytical sensitivity (*γ* = 1.05 mL ng^−1^, *γ*^−1^ = 0.96 ng mL^−1^) enabled exceptional quantification at trace levels. Limits of detection (LOD) and quantitation (LOQ) were 119.87 and 363.23 ng mL^−1^ for NOR and 3.16 and 9.56 ng mL^−1^ for MET. Furthermore, with reference to ICH M10 bioanalytical validation guidance [47,48], donor-to-donor matrix variability was evaluated across pre-dose urine specimens obtained from the five individual healthy volunteers. Mean relative recoveries for spiked matrix lots fell well within 94.2–104.6% with RSD < 4.8%, proving that the second-order advantage successfully modeled and insulated analyte signals against inter-individual endogenous fluorophore variations. Lorber’s selectivity (SEL) [50] measures the fraction of the analyte’s spectral signal that is fully orthogonal to the subspace spanned by all co-existing interferents (urine autofluorescence and NOR emission). An SEL value of 0.15 indicates that 85% of MET’s total variance overlaps with the background matrix, leaving 15% as net analytical signal (NAS). While PARAFAC’s second-order advantage mathematically enables deconvolution even at low selectivity, an SEL of 0.15 increases the condition number of the loading inner-product matrix, making the system more vulnerable to noise amplification when matrix background fluctuates abnormally. Under normal physiological conditions, endogenous urinary fluorophores (e.g., pteridines and pyridoxic acid [23]) fluctuating up to 2.5× baseline are seamlessly isolated into the background factor without distorting MET loadings (bias < 4.5%). However, the method reaches an upper tolerance limit when matrix fluorescence exceeds ~5× baseline levels, where photon shot noise begins to erode the 15% orthogonal subspace. Specifically, the method reaches its failure boundary under: (1) severe hematuria or hyperbilirubinemia (jaundice), where high concentrations of free hemoglobin or bilirubin produce strong absorption at 400–420 nm (Soret band), inducing non-linear inner-filter reabsorption and breaking trilinearity; (2) massive proteinuria (>3 g L^−1^), where high albumin/globulin levels cause colloidal scattering and hydrophobic binding interactions; and (3) co-administration of highly fluorescent drugs with overlapping emission (e.g., triamterene, doxorubicin). When such abnormal biological specimens are encountered, trilinearity and analytical reliability can be readily restored by increasing the dilution factor from 10-fold to 20-fold or 50-fold, or by passing the sample through a 0.22 µm PTFE syringe membrane filter prior to spectrofluorimetric reading. Furthermore, matrix effects and freeze–thaw stability were evaluated following ICH M10 recommendations [47]. Matrix Factor (MF) was assessed across blank urine specimens obtained from six individual donors (Table S4). The mean absolute MFs were 1.012 (inter-lot %CV = 3.24%) and 0.994 (%CV = 2.46%) for NOR at Low QC and High QC, respectively, and 0.968 (%CV = 3.97%) and 0.985 (%CV = 2.45%) for MET. All %CV values were well below the ICH M10 threshold of ≤15%, demonstrating that matrix-induced fluorescence quenching or enhancement is negligible. Additionally, three-cycle freeze–thaw stability testing (−20 °C to room temperature) yielded mean recoveries of 96.40–101.65% with %RSD ≤ 4.82% and intact CORCONDIA (>98%, Table S5), verifying that frozen clinical urine specimens retain full spectral and quantitative stability.

### 2.4. Application to Human Urinary Excretion Profiles

**Figure 5:**
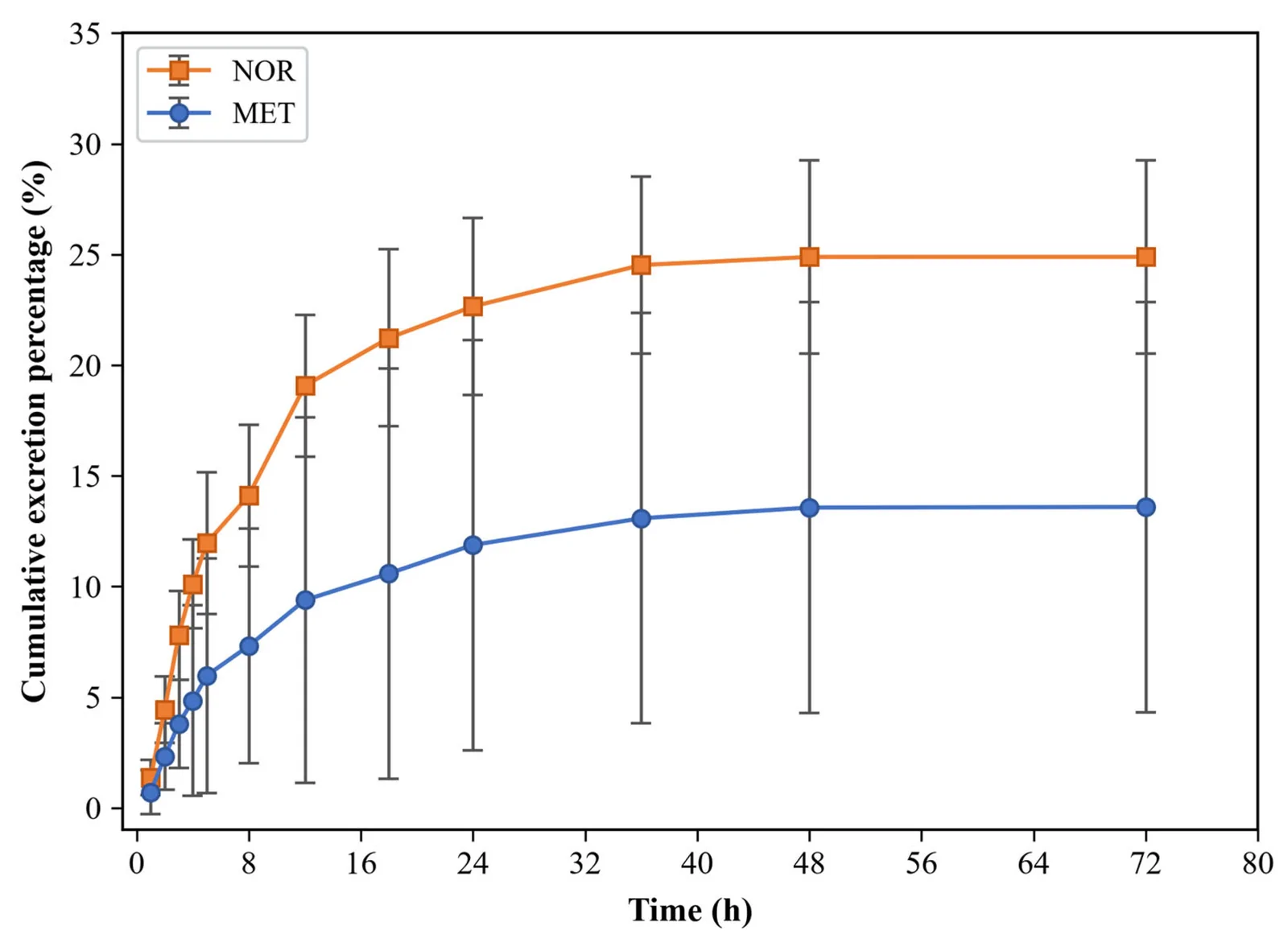
Mean urinary cumulative excretion percentage–time profiles of NOR (400 mg) and MET (10 mg) in urine donors (*n* = 5, 0–72 h). Data are represented as Mean ± SD (bidirectional error bars).

**Figure 6:**
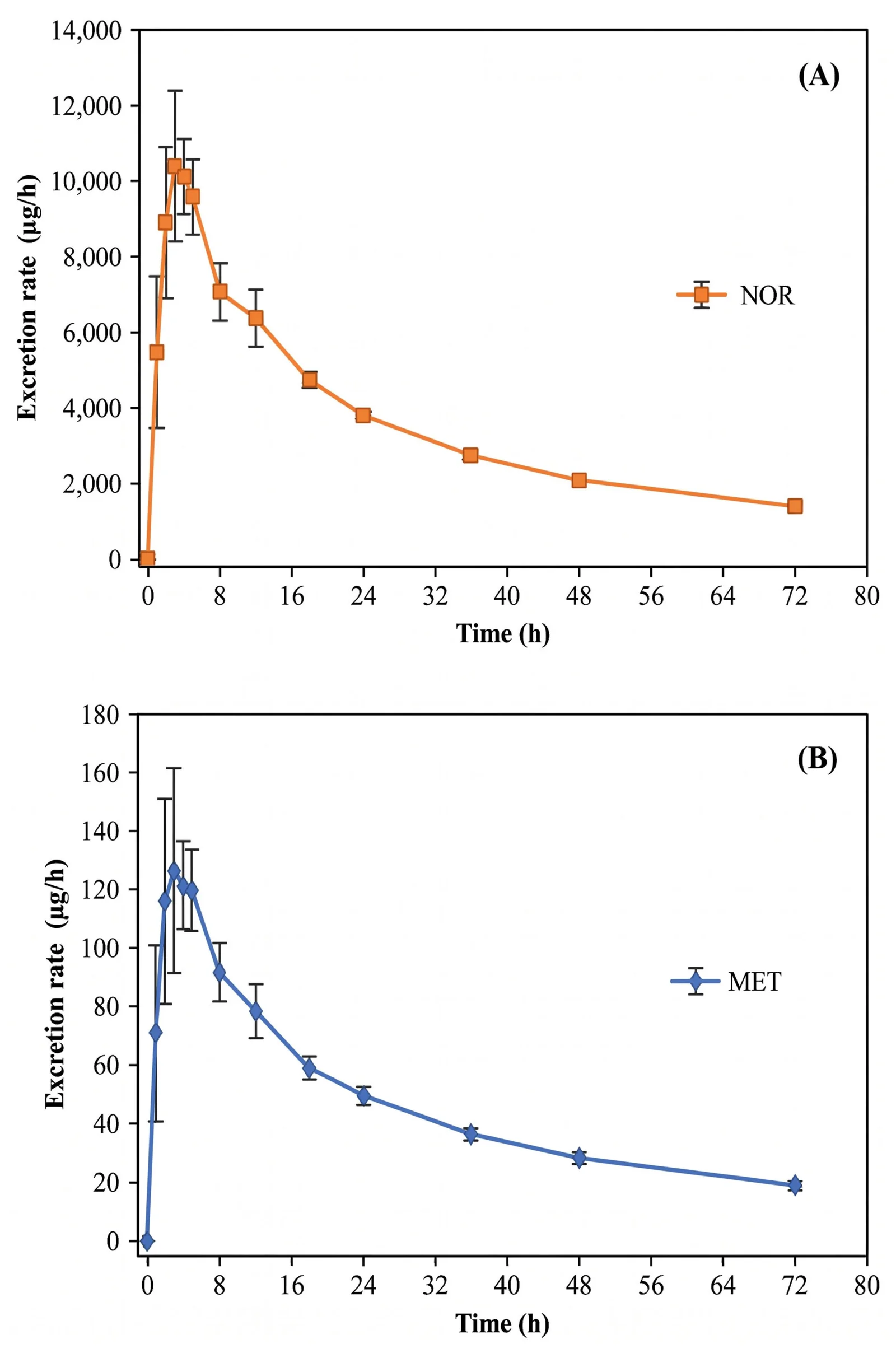
Mean urinary excretion rate–time profiles of (**A**) NOR (µg h^−1^) and (**B**) MET (µg h^−1^) in urine donors (*n* = 5). Data are represented as Mean ± SD (bidirectional error bars).

The validated dual-submatrix PARAFAC method was applied to urine samples collected from five healthy volunteers over a 72-h period following oral co-administration of Noracin^®^ (400 mg NOR) and Primperan^®^ (10 mg MET) tablets. Mean cumulative urinary excretion percentage–time curves and urinary excretion rate–time curves are presented in Figure 5 and Figure 6, respectively. The complete interval-by-interval excretion data, urinary volumes, drug concentrations, and excretion rates for individual volunteers are detailed in Table S6 (for NOR) and Table S7 (for MET), with cumulative percentage recovery profiles tabulated in Table S8. For both drugs, maximum urinary excretion rates were achieved within the initial 1–3 h post-dosing intervals, corresponding to the expected absorption and distribution phases: the NOR excretion rate peaked at 13.38 ± 1.85 mg h^−1^ (midpoint 2.5 h), whereas the MET excretion rate peaked at 160.84 ± 18.25 µg h^−1^ (midpoint 1.5 h). Excretion declined rapidly thereafter, with the process being virtually complete by 48 h. Cumulative 72-h urinary excretion amounted to 116.54 ± 18.51 mg for NOR (representing 24.89 ± 4.63% of the 400 mg dose) and 1.74 ± 0.92 mg for MET (representing 13.59 ± 9.27% of the 10 mg dose). These urinary recoveries can be compared with published pharmacokinetic data for NOR [8] and MET [6]; however, because no chromatographic reference analysis was performed on the volunteer samples, the present data do not by themselves establish quantitative agreement with chromatographic methods.

### 2.5. Multi-Metric Green, White, and Blue Analytical Chemistry Assessment

**Figure 7:**
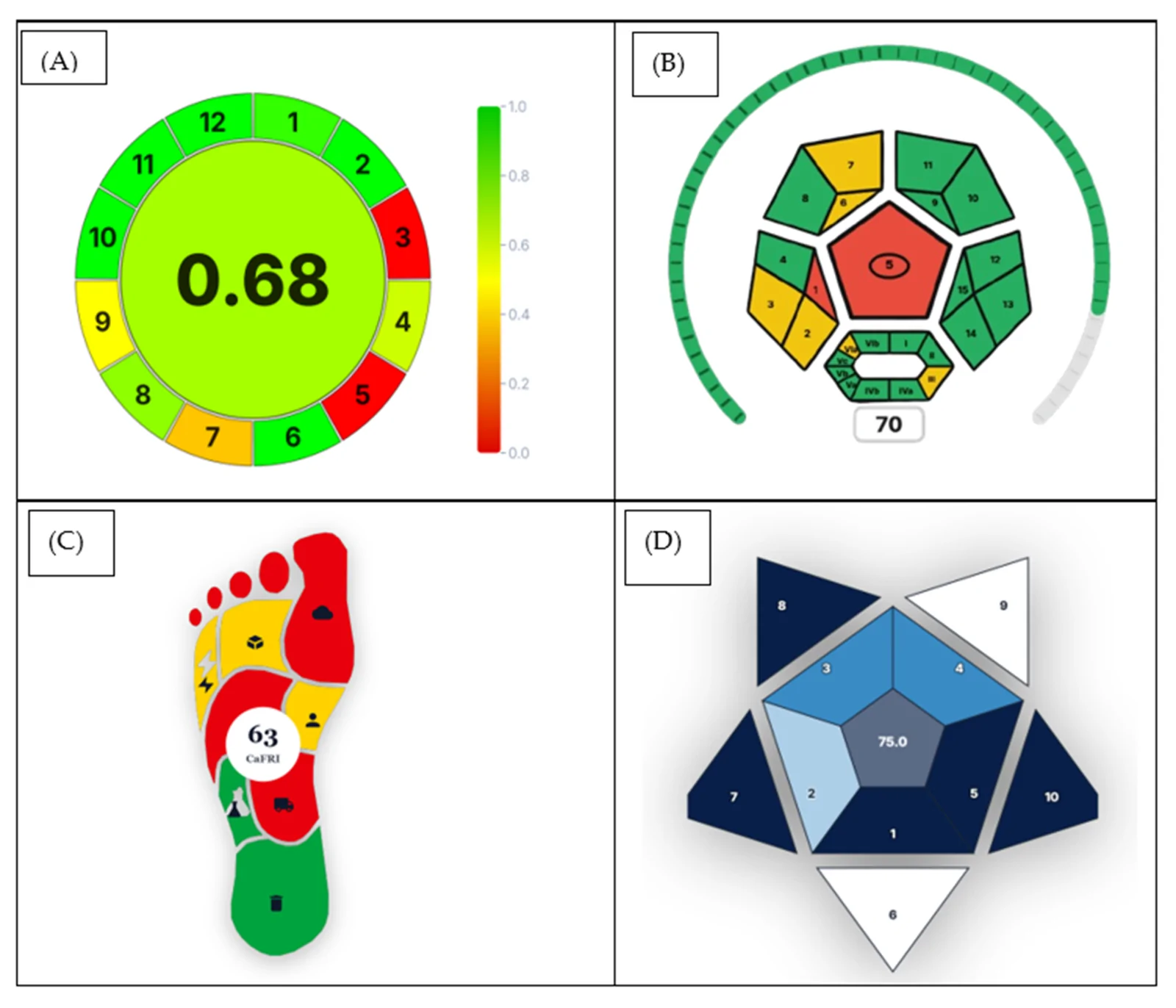
Visual outputs of the greenness and applicability assessment tools applied to the proposed method: (**A**) AGREE pictogram, (**B**) ComplexMOGAPI pictogram, (**C**) CaFRI output, and (**D**) BAGI pictogram.

To establish the sustainability and operational profile of the developed procedure, four modern evaluation tools were applied: AGREE [25], ComplexMoGAPI [26], CaFRI [27], and BAGI [49]. The corresponding assessment pictograms are depicted in Figure 7, and the comprehensive parameter input criteria, scoring breakdowns, and penalty evaluations are presented in Table S9. AGREE, which scores compliance across the 12 principles of Green Analytical Chemistry on a 0–1 scale, assigned the method a score of 0.68 (Figure 7A). Favorable contributions derived from the direct measurement in minimally prepared urine, absence of hazardous extraction solvents, ambient operation, and low energy demand; penalties were limited to manual offline sampling and multi-analyte sequential calibration. ComplexMoGAPI, which integrates greenness and occupational safety, yielded a high score of 69 (‘Excellent Greenness’, Figure 7B), reflecting the minimal waste generation (<2 mL aqueous buffer per test) and complete elimination of flammable, toxic organic solvents (acetonitrile, methanol). The Carbon Footprint Reduction Index (CaFRI) assigned a score of 63.1 (‘Average/Yellow’, Figure 7C), highlighting the intrinsically low carbon footprint of benchtop spectrofluorimetry compared to high-power mass spectrometers, while identifying future opportunities for automated inline processing. BAGI, which evaluates practical applicability and operational simplicity, awarded the procedure a score of 75.0 (Figure 7D), denoting outstanding routine utility due to standard cuvette instrumentation, zero sample extraction, and rapid analysis time (~1 min per EEM scan). To contextualize these findings, Table 6 benchmarks the proposed EEM-PARAFAC method against reported HPLC, HPLC-FLD, and LC-MS/MS bioanalytical procedures for NOR and MET. The comparison reveals that the proposed method eliminates 100% of organic solvent consumption (saving 15–50 mL per sample), slashes sample preparation time from 30 min to 2 min, requires zero expensive chromatographic columns, and achieves markedly superior greenness (AGREE: 0.68 vs. 0.42–0.51) and practicality scores (BAGI: 75.0 vs. 52.0–61.0). Practical implementation and clinical laboratory translation: Notwithstanding its overwhelming greenness and speed advantages over LC-MS/MS, translation of the EEM-PARAFAC method into routine hospital and commercial clinical pathology laboratories faces practical adoption barriers that warrant consideration. First, conventional clinical laboratories are predominantly geared toward automated multi-channel chemistry analyzers and automated immunoassays, whereas scanning 3D spectrofluorimeters are less universally available in routine wards. Nonetheless, a benchtop spectrofluorimeter incurs an order-of-magnitude lower capital investment (~$15,000–$30,000 vs. >$250,000 for LC-MS/MS), requires no expensive chromatographic columns or ultra-pure mobile phases, and generates zero toxic solvent waste, making it highly accessible for decentralized or resource-limited settings. Second, a key operational barrier is the chemometric programming threshold: routine laboratory personnel lack specialized expertise in Python, MATLAB, or tensor factorization algorithms. To bridge this gap, routine implementation can be achieved by deploying compiled standalone graphical user interface (GUI) software wrappers or automated cloud-based processing pipelines. Frontline operators simply import the raw EEM data file and receive instant concentration readouts based on locked, pre-calibrated PARAFAC loading models. Furthermore, adapting the protocol to 96-well microplate spectrofluorimeters equipped with automated monochromators will facilitate seamless integration into high-throughput clinical laboratory robotics.

## 3. Experimental Section

### 3.1. Instrumentation

Fluorescence measurements were performed using a Perkin-Elmer (Beaconsfield, UK) model LS-45 luminescence spectrometer equipped with a 150 W Xenon arc lamp, excitation and emission holographic grating monochromators, and a photomultiplier detector operated at 800 V. Slit widths for both excitation and emission monochromators were maintained constant at 10 nm. A standard 1.0 cm pathlength quartz fluorescence cuvette was employed. Instrument control and spectral data collection were executed using FL WinLab software (Version 4.00.02, Perkin-Elmer, Beaconsfield, UK). The spectral bandwidth was set to 0.5 nm, and the wavelength scanning speed was 1500 nm min^−1^.

### 3.2. Reagents and Pharmaceutical Formulations

Pharmaceutical-grade reference standards of norfloxacin (purity 99.1% *w*/*w* on dried basis) and metoclopramide hydrochloride (purity 99.5% *w*/*w* on dried basis) were kindly supplied by the Egyptian International Pharmaceutical Industries Company (EIPICO, 10th of Ramadan City, Egypt) and Tabuk Pharmaceuticals (Kingdom of Saudi Arabia), respectively.

HPLC-grade formic acid, phosphoric acid, ethanol, and methanol were purchased from Fisher Scientific Ltd. (Loughborough, UK). Sodium hydroxide and sodium dihydrogen phosphate dihydrate were purchased from Loba Chemie Pvt. Ltd. (Mumbai, India). Deionized water purified via a Milli-Q system (Millipore, Bedford, MA, USA) was used throughout for buffer and solution preparation.

Two commercial solid dosage forms were administered to healthy volunteers in the excretion study: Noracin^®^ tablets (Memphis Pharmaceutical & Chemical Industry, Egypt; Batch No. 314229, labeled to contain 400 mg NOR per tablet) and Primperan^®^ tablets (Sanofi Aventis Egypt s.a.e.; Batch No. 6EG005, labeled to contain 10 mg MET per tablet). Both commercial products were procured from local community pharmacies.

### 3.3. Preparation of Stock and Working Standard Solutions

Primary stock standard solutions of NOR and MET (500.0 µg mL^−1^ each) were prepared by accurately weighing 25.0 mg of each pure reference standard. NOR was dissolved in the minimum volume of formic acid (~100 µL) to facilitate dissolution, transferred quantitatively into a 50.0 mL volumetric flask, and brought to volume with 0.1 M phosphate buffer (pH 3.0). MET was dissolved directly in 0.1 M phosphate buffer (pH 3.0) and diluted to 50.0 mL. Intermediate working standard solutions (10.0 µg mL^−1^ for both drugs) were prepared by appropriate serial dilution of primary stocks with 0.1 M phosphate buffer (pH 3.0). Standard working mixtures and spiked biological calibration samples were subsequently prepared by volumetric transfer into 0.1 M phosphate buffer (pH 3.0) to achieve the finalized linear dynamic calibration ranges of 1000–6000 ng mL^−1^ for NOR and 15–80 ng mL^−1^ for MET (Table 1), reflecting the vast biological concentration disparity expected post-administration.

Phosphate buffer (0.1 M, pH 3.0) was chosen as the operational solvent because it maximizes the native fluorescence intensity of MET while maintaining excellent NOR signal stability, without requiring hazardous organic co-solvents. Furthermore, 10-fold dilution was adopted as the standardized sample preparation step, providing optimal attenuation of urine background autofluorescence while keeping the minor analyte (MET) comfortably above its quantification threshold.

### 3.4. Experimental Design and Volunteer Urinary Excretion Protocol

#### 3.4.1. Calibration and Validation Spiked Urine Sets

Drug-free blank urine matrices were pooled from drug-free healthy human donors and centrifuged at 3000 rpm (1000× *g*) for 10 min to remove cellular debris. To construct the multiway calibration model, a 10-sample orthogonal/randomized calibration set and a 5-sample independent external validation set were prepared in urine as specified in Table 1. Each sample was prepared by spiking appropriate volumes of intermediate standard solutions into 1.0 mL of centrifuged urine in a 10.0 mL volumetric flask, followed by dilution to volume with 0.1 M phosphate buffer (pH 3.0), yielding a standardized 10-fold biological matrix dilution. Two pure reference samples of each drug were also analyzed to establish reference spectral loadings. To evaluate biological matrix effects following the ICH M10 guideline [47], drug-free blank urine specimens were also collected from six individual healthy human donors (specific gravity ranging from 1.008 to 1.028). For each donor lot, matrix factor (MF) testing was conducted by spiking Low QC and High QC concentrations of NOR and MET into post-diluted blank urine and comparing the resolved sample loadings against equivalent neat standard solutions in buffer. Furthermore, sample freeze–thaw stability was evaluated across three consecutive freeze–thaw cycles (−20 °C to room temperature, ≥24 h per cycle) at Low and High QC levels (*n* = 5 replicates per level) to verify the preservation of trilinear fluorescence profiles and mathematical deconvolution fidelity under standard bioanalytical storage conditions. All calibration mixtures (*n* = 10, spanning 1500–6000 ng mL^−1^ for NOR and 20–80 ng mL^−1^ for MET) and external validation mixtures (*n* = 5) were prepared and measured in triplicate (*n* = 3 independent biological replicates per concentration level). Validation mixtures containing 1000 ng mL^−1^ NOR (Mixture 3) and 15 ng mL^−1^ MET (Mixture 1) were specifically evaluated as lower-boundary challenge samples situated between the lowest calibration level and the limit of quantitation to assess model stability under boundary stress.

#### 3.4.2. Human Volunteer Urinary Excretion Study Protocol

The urinary excretion study was conducted to demonstrate the direct applicability of the validated PARAFAC method to real biological samples. The clinical protocol was reviewed and formally approved by the Research Ethics Committee at the Faculty of Pharmacy, Suez Canal University (Ethical Approval No. 201608RH1). The study was executed in strict compliance with the Declaration of Helsinki and institutional ethical standards. Five healthy adult male volunteers (aged 24–30 years, mean weight 74 ± 6 kg) participated after providing written informed consent. All participants were confirmed to be non-smokers, had normal renal and hepatic functions, and abstained from medications for at least two weeks prior to the study. Following an overnight fast of 10 h, each volunteer orally ingested one Noracin^®^ tablet (400 mg NOR) and one Primperan^®^ tablet (10 mg MET) with 250 mL of water. Complete fluid restriction was observed for the first 2 h post-dosing to standardize gastric emptying and absorption kinetics. Between 2 h and 24 h post-dosing, fluid intake was regulated according to a standardized hydration protocol: volunteers consumed 150–200 mL of water every 2 h during daytime waking hours to maintain consistent physiological diuresis (1.0–2.0 mL min^−1^) and avoid bladder retention. All fluid intake was logged in Case Report Forms (total daily intake: 2200 ± 250 mL). Standardized meals were provided at 4 h and 10 h post-dosing. Because urinary pharmacokinetic calculations (Equations (1)–(3)) evaluate absolute drug mass excreted (Δ*X*_u_ = *C*_urine_ × *V*_urine_) and mass excretion rate (d*D*_u_/d*t* = Δ*X*_u_/Δ*t*), biological variations in voided volume and dilution naturally compensate, ensuring complete physiological comparability across volunteers. Pre-dose blank urine samples (0 h) were collected, followed by complete urine collection across 12 timed post-dose intervals: 0–1, 1–2, 2–3, 3–4, 4–5, 5–8, 8–12, 12–18, 18–24, 24–36, 36–48, and 48–72 h. Urine volume was recorded at each interval, and 20.0 mL aliquots were stored at −20 °C until analysis. Before spectrofluorimetric acquisition, samples were thawed at room temperature, centrifuged at 3000 rpm (1000× *g*) for 10 min, diluted 10-fold with 0.1 M phosphate buffer (pH 3.0), and measured directly without extraction or filtration.

#### 3.4.3. Excretion Parameters and Pharmacokinetic Calculations

Urinary drug concentrations were computed directly from PARAFAC model sample loadings. Urinary excretion parameters were calculated according to Equations (1)–(4):

*Excretion amount* (mg) *= C*_urine_
*× V*_urine_ (1)

*Excretion rate* (mg h^−1^) *= Excretion amount/*Δ*t* (2)

*Cumulative excretion amount* (mg) *= ∑ (Excretion amount)_i_* (3)

*Cumulative excretion percentage (%) = (Cumulative excretion amount/Administered Dose) × 100%* (4)

where *C*_urine_ is the measured drug concentration (converted to mg mL^−1^ after correcting for the 10-fold dilution factor), *V*_urine_ is the total urine volume excreted during interval Δ*t*, and Administered Dose is 400 mg for NOR and 10 mg for MET.

#### 3.4.4. Chemometric Modeling and Computational Software

All chemometric routines, data array manipulations, and PARAFAC decompositions were executed in MATLAB^®^ Version 7.10.0 (R2010a, MathWorks Inc., Natick, MA, USA) utilizing the N-way toolbox (Version 3.1; Rasmus Bro and Claus A. Andersson, University of Copenhagen, Frederiksberg, Denmark) [51] alongside custom in-house scripts for multivariate figures of merit and greenness metrics calculations.

### 3.5. Mathematical Foundation of PARAFAC and Model Diagnostics

Parallel factor analysis (PARAFAC) decomposes a three-way data array **X** of dimensions *I* (samples) × *J* (emission wavelengths) × *K* (excitation wavelengths) into *F* trilinear components according to Equation (5) [33]:

*x_ijk_ = ∑_f=_*_1_*^F^ a_if_ · b_jf_ · c_kf_ + e_ijk_* (5)

where *x_ijk_* is the fluorescence intensity of sample *i* at emission variable *j* and excitation variable *k*; *a_if_*, *b_jf_*, and *c_kf_* represent elements of the loading matrices **A** (*I* × *F*, sample concentrations), **B** (*J* × *F*, emission spectral profiles), and **C** (*K* × *F*, excitation spectral profiles), respectively; and *e_ijk_* represents the unmodeled residual array **E** (*I* × *J* × *K*). Alternating least squares (ALS) with non-negativity constraints in all three modes was implemented to guarantee physically realistic, non-negative spectral loadings and concentrations.

The optimal number of factors (*F*) was established using the Core Consistency Diagnostic (CORCONDIA) [35], percentage of explained variance (%Fit), and inspection of residual spectral distributions. Models exhibiting CORCONDIA > 85% were deemed trilinear and structurally sound, whereas models with CORCONDIA < 50% were rejected as overparameterized. Component identification was confirmed by cross-correlating recovered spectral loadings (**B** and **C**) with authentic pure standard fluorescence spectra.

### 3.6. Multivariate Figures of Merit (FOM)

To validate method performance in accordance with IUPAC recommendations and second-order calibration theory [50,52], figures of merit were derived from the Net Analyte Signal (NAS)—the portion of the instrumental response uniquely attributable to the analyte and orthogonal to all interfering contributions.

Sensitivity (SEN*_n_*) for analyte *n* represents the net instrumental response generated per unit concentration under the modeled matrix interference:

*SEN_n_ = s_n_ · [**b**_n_^T^ · **P_B_**_,−n_^⊥^ · **b**_n_]^1/2^ · [**c**_n_^T^ · **P_C_**_,−n_^⊥^ · **c**_n_]*^1/2^ (6)

where *s_n_* is the calibration slope relating sample loadings (*a_n_*) to analyte concentration, **b***_n_* and **c***_n_* are normalized emission and excitation loadings, and **P_B_**_,−*n*_^⊥^ and **P_C_**_,−*n*_^⊥^ are orthogonal projection matrices that remove the overlap of background and co-analyte fluorophores in each mode.

Selectivity (SEL*_n_*) quantifies the fraction of the analyte’s total fluorescence signal that remains available for quantification after accounting for matrix and co-analyte overlap:

*SEL_n_ = SEN_n_/s_n_ = [**b**_n_^T^ · **P_B_**_,−n_^⊥^ · **b**_n_]^1/2^ · [**c**_n_^T^ · **P_C_**_,−n_^⊥^ · **c**_n_]*^1/2^ (7)

Analytical sensitivity (*γ_n_*) incorporates instrumental and matrix noise (*s_x_*), reflecting the concentration discrimination capability of the system. Its reciprocal (*γ_n_*^−1^) defines the minimum concentration difference distinguishable between two samples at 95% confidence:

*γ_n_ = SEN_n_/s_x_; γ_n_*^−1^ *= s_x_/SEN_n_* (8)

Limits of detection (LOD) and quantitation (LOQ) were computed in accordance with Lorber and Faber–Olivieri formalisms [50,52] based on the standard deviation of blank biological replicates (*s*_0_):

*LOD_n_ = 3.3 · (s_0_/SEN_n_) = 3.3 · s_0_ · γ_n_*^−1^ (9)

*LOQ_n_ = 10 · (s_0_/SEN_n_) = 10 · s_0_ · γ_n_*^−1^ (10)

## 4. Conclusions

In this study, a dual-submatrix spectral partitioning strategy (**X**_NOR_ and **X**_MET_) addressed the difficulty of resolving analytes with large concentration imbalances in the presence of biological autofluorescence. Norfloxacin and metoclopramide were reliably quantified in human urine across a 40:1 to 75:1 concentration disparity after a simple 10-fold dilution with pH 3.0 aqueous buffer, completely eliminating toxic organic solvents, solid-phase extraction, and chromatographic separation, in line with current advances in green sample preparation for drug bioanalysis [24]. The method was validated with reference to ICH M10 principles [47], demonstrated robustness across individual human urine matrices, and successfully tracked 72-h urinary excretion kinetics in healthy volunteers following oral administration of commercial Noracin^®^ (400 mg) and Primperan^®^ (10 mg) tablets. Multi-tool sustainability assessment (AGREE: 0.68, ComplexMoGAPI: 69, CaFRI: 63.1, BAGI: 75.0) confirmed substantial ecological, economic, and operational advantages over conventional HPLC and LC-MS/MS platforms. This work revitalizes PARAFAC as an indispensable, ultra-green tool for pharmaceutical bioanalysis and quality control. Looking forward, the dual-submatrix mathematical chromatography paradigm opens several compelling avenues for future research and technological maturation: (1) Robotic and Flow-Injection Automation: Interfacing the minimal-preparation protocol with automated flow-injection manifolds or robotic liquid handlers coupled to rapid-scanning microplate spectrofluorimeters will elevate sample throughput to hundreds of analyses per hour, paving the way for real-time therapeutic drug monitoring (TDM); (2) Advanced Chemometric Algorithms: Exploring non-linear or shift-tolerant multiway algorithms—such as PARAFAC2 (to accommodate minor matrix-induced spectral shifts), Alternating Trilinear Decomposition (ATLD), and physics-informed neural network tensor factorizations—promises to further suppress background fluctuations, lower the limit of detection (LOD) to the sub-nanogram-per-milliliter regime, and improve resilience in low-selectivity regimes; (3) Extension to Complex Biological Matrices: Adapting this green dilute-and-shoot strategy to more protein-dense biological fluids, including plasma, serum, and saliva, via biocompatible micellar-enhanced fluorimetry or sub-milliliter green protein precipitation will establish mathematical chromatography as a universal, low-cost analytical platform across preclinical and clinical pharmacokinetics.

## Figures and Tables

**Table 1:** Experimental design of calibration (10 mixtures) and external validation (5 mixtures) spiked urine samples (ng mL^−1^).

Mixture No.	Calibration NOR (ng mL^−1^)	Calibration MET (ng mL^−1^)	Validation NOR (ng mL^−1^)	Validation MET (ng mL^−1^)
1	3000	40	5000	15
2	6000	60	2000	50
3	4500	40	1000	70
4	3000	80	3500	35
5	2250	40	4000	45
6	3000	60	—	—
7	1500	40	—	—
8	6000	20	—	—
9	6000	80	—	—
10	3000	20	—	—

**Table 2:** Quantitative prediction results for the calibration set in human urine by the dual-submatrix PARAFAC models.

Sample No.	Added NOR (ng mL^−1^)	Found NOR (ng mL^−1^)	Recovery NOR (%)	Added MET (ng mL^−1^)	Found MET (ng mL^−1^)	Recovery MET (%)
1	3000.00	3074.39	102.48	40.00	39.75	99.38
2	6000.00	6001.26	100.02	60.00	58.97	98.29
3	4500.00	4536.55	100.81	40.00	39.74	99.35
4	3000.00	2997.95	99.93	80.00	79.09	98.86
5	2250.00	2226.78	98.97	40.00	39.73	99.32
6	3000.00	3008.89	100.30	60.00	59.54	99.23
7	1500.00	1432.20	95.48	40.00	42.74	106.85
8	6000.00	5963.44	99.39	20.00	19.76	98.79
9	6000.00	5972.14	99.54	80.00	81.07	101.33
10	3000.00	3036.40	101.21	20.00	19.62	98.09
Mean Recovery (%)	—	—	99.81	—	—	99.95
Standard Deviation (S.D., %)	—	—	1.83	—	—	2.58
RMSEC (ng mL^−1^)	—	70.77	—	—	0.86	—

**Table 3:** Quantitative prediction results and overall statistical validation for the external test set in human urine by PARAFAC models.

Sample No.	Added NOR (ng mL^−1^)	Found NOR (ng mL^−1^)	Recovery NOR (%)	Added MET (ng mL^−1^)	Found MET (ng mL^−1^)	Recovery MET (%)
1	5000.00	5080.49	101.61	15.00	14.67	97.82
2	2000.00	2048.45	102.42	50.00	53.38	106.76
3	1000.00	1057.70	105.77	70.00	72.93	104.19
4	3500.00	3528.73	100.82	35.00	34.13	97.52
5	4000.00	3734.06	93.35	45.00	46.35	103.00
Mean Recovery (%)	—	—	100.79	—	—	101.86
Standard Deviation (S.D., %)	—	—	4.57	—	—	4.06
RMSEP (ng mL^−1^)	—	144.65	—	—	2.38	—

**Table 4:** Chemometric modeling parameters and algorithmic diagnostics for PARAFAC deconvolution of NOR and MET.

Chemometric Parameter/Diagnostic	Norfloxacin Sub-Array (XNOR)	Metoclopramide Sub-Array (XMET)
Excitation range (λ_ex_, nm)	260–310 nm (26 variables, step 2 nm)	260–310 nm (26 variables, step 2 nm)
Emission range (λ_em_, nm)	440–500 nm (111 variables, step 0.5 nm)	380–410 nm (61 variables, step 0.5 nm)
Array dimensions (*I* × *J* × *K*)	16 × 111 × 26	16 × 61 × 26
Number of components (*F*)	2 (NOR + urine matrix)	3 (MET + residual NOR tail + urine)
Model constraints	Non-negativity in all three modes	Non-negativity in all three modes
Core Consistency Diagnostic (CORCONDIA, %)	98.7%	94.2%
Explained variance (%Fit)	99.92%	99.85%
Root mean square error of calibration (RMSEC, ng mL^−1^)	70.77	0.86
Root mean square error of prediction (RMSEP, ng mL^−1^)	144.65	2.38
Correlation coefficient (*R*^2^)	0.9981	0.9995

**Table 5:** Multivariate figures of merit for determination of NOR and MET in human urine by PARAFAC models.

Parameter	SEN	SEL	γ (mL ng^−1^)	γ^−1^ (ng mL^−1^)	LOD (ng mL^−1^)	LOQ (ng mL^−1^)
NOR ^a^	1.12	0.42	0.03	36.32	119.87	363.23
MET ^a^	1.96	0.15	1.05	0.96	3.16	9.56

^a^ Values expressed are sample-specific and represent the mean across the entire biological calibration set in human urine.

**Table 6:** Comparison of the proposed EEM-PARAFAC method with reported chromatographic methods for the bioanalysis of NOR and MET.

Analytical Method	Target Analytes	Biological Matrix	Sample Pretreatment	Organic Solvents Consumed	Analysis Run Time	AGREE Score	BAGI Score
Proposed EEM-PARAFAC	NOR + MET	Human urine	10-fold dilution (buffer)	0.0 mL (100% aqueous)	~1 min/sample	0.68	75.0
Reported HPLC [12,20]	NOR or MET	Urine/Serum	Solid-phase/liquid extraction	15–30 mL (Methanol/ACN)	12–18 min	0.46	58.0
Reported HPLC-FLD [11]	NOR	Plasma/Urine	Protein precipitation/SPE	10–25 mL (ACN/Phosphate)	15 min	0.51	61.0
Reported LC-MS/MS [21,22]	MET	Human plasma	SPE with C18 cartridge	20–40 mL (Methanol/ACN)	8–12 min	0.42	52.0

## Data Availability

The data presented in this study are available on request from the corresponding author.

## Supplementary Materials

The following supporting information can be downloaded at: https://www.mdpi.com/article/10.3390/molecules31213984/s1, Table S1: Timed urinary excretion profiles and excretion rates of Norfloxacin (NOR) in healthy human volunteers (*n* = 5) following oral administration of Noracin® tablets (400 mg NOR); Table S2: Timed urinary excretion profiles and excretion rates of Metoclopramide (MET) in healthy human volunteers (*n* = 5) following oral administration of Primperan® tablets (10 mg MET); Table S3: Mean cumulative urinary excretion percentage (%) and cumulative mass recovered over 72 h in human volunteers; Table S4: Factor selection diagnostics: Core Consistency Diagnostic (CORCONDIA %) and percentage of explained variance (%Fit) as a function of the number of factors (F); Table S5: Calibration curve regression parameters for PARAFAC sample-mode loadings (a_if) vs. nominal drug concentrations in spiked human urine; Table S6: Parameter breakdown and scoring profiles for multi-metric sustainability and applicability tools; Table S7: Freeze–thaw stability evaluation of Norfloxacin (NOR) and Metoclopramide (MET) in spiked human urine across 3 consecutive cycles (*n* = 5; exploratory below-range NOR results included); Table S8: Quantitative performance and chemometric diagnostic comparison between Proposed Dual-Submatrix PARAFAC, Traditional Global PARAFAC, and Second-Order Standard Addition Method (SOSAM) for NOR and MET in human urine; Table S9: Assessment of matrix-associated response ratios (Matrix Factor, MF) across 6 individual human urine donor lots.
