# Supplementary material for: PARAFAC-Assisted Excitation–Emission Matrix Fluorescence for Minimal-Preparation Co-Determination of Norfloxacin and Metoclopramide in Human Urine: Overcoming Severe Concentration Imbalance Without Chromatography
Source: Molecules. 2026 Nov 4;31(21):3984. doi: 10.3390/molecules31213984 (PMC13764746; doi:10.3390/molecules31213984)
Supplement: Supplementary file 1 [file molecules-31-03984-s001.zip › molecules-4647466-supplementary.pdf]

Supplementary Materials

PARAFAC-Assisted Excitation–Emission Matrix Fluorescence for Minimal-Preparation Co-Determination of Norfloxacin and Metoclopramide in Human Urine: Overcoming Severe Concentration Imbalance Without Chromatography

Mahmoud M. Elkhoudary [1,2,*], Randa A. Abdel Salam [3], Mohamed S. Abdel-Maksoud [4] and Ghada M. Hadad [3]

1 Department of Pharmaceutical Chemistry, Faculty of Pharmacy, Horus University-Egypt, New Damietta 34518, Egypt
2 Department of Pharmaceutical Analytical Chemistry, Faculty of Pharmacy, East Port Said National University, Port Said 42526, Egypt
3 Department of Pharmaceutical Analytical Chemistry, Faculty of Pharmacy, Suez Canal University, Ismailia 41522, Egypt
4 Department of Pharmacology and Toxicology, Faculty of Pharmacy, University of Tabuk, Tabuk 71491, Saudi Arabia
** Correspondence: melkhodary@horus.edu.eg; Tel.: +20-1110777922*

Table S1. Timed urinary excretion profiles and excretion rates of Norfloxacin (NOR) in healthy human volunteers (n = 5) following oral administration of Noracin® tablets (400 mg NOR).

Collection Interval (h)	Midpoint (h)	Urine Volume (mL)	Measured Conc. (µg mL^{-1})	Excretion Amount (mg)	Excretion Rate (mg h^{-1})
0–1	0.5	203.0 ± 24.5	26.89 ± 3.45	5.46 ± 0.82	5.46 ± 0.82
1–2	1.5	269.3 ± 31.2	45.78 ± 5.12	12.33 ± 1.64	12.33 ± 1.64
2–3	2.5	267.3 ± 28.6	50.06 ± 6.30	13.38 ± 1.85	13.38 ± 1.85
3–4	3.5	143.6 ± 19.8	64.62 ± 7.42	9.28 ± 1.25	9.28 ± 1.25
4–5	4.5	331.7 ± 42.1	22.30 ± 3.10	7.40 ± 0.98	7.40 ± 0.98
5–8	6.5	163.4 ± 22.0	52.70 ± 5.80	8.61 ± 1.15	2.87 ± 0.38
8–12	10.0	508.9 ± 55.4	38.97 ± 4.25	19.83 ± 2.45	4.96 ± 0.61
12–18	15.0	339.6 ± 38.2	25.39 ± 3.12	8.62 ± 1.18	1.44 ± 0.20
18–24	21.0	227.6 ± 26.5	25.06 ± 2.95	5.70 ± 0.82	0.95 ± 0.14
24–36	30.0	1580.2 ± 145.0	4.73 ± 0.65	7.47 ± 1.05	0.62 ± 0.09
36–48	42.0	1422.8 ± 132.5	1.03 ± 0.18	1.47 ± 0.28	0.12 ± 0.02
48–72	60.0	2150.0 ± 180.2	Trace (<LOQ)	Trace	—

Data are represented as Mean ± SD across 5 healthy adult male volunteers.

Table S2. Timed urinary excretion profiles and excretion rates of Metoclopramide (MET) in healthy human volunteers (n = 5) following oral administration of Primperan® tablets (10 mg MET).

Collection Interval (h)	Midpoint (h)	Urine Volume (mL)	Measured Conc. (ng mL^{-1})	Excretion Amount (µg)	Excretion Rate (µg h^{-1})
0–1	0.5	203.0 ± 24.5	348.8 ± 42.1	70.79 ± 9.54	70.79 ± 9.54
1–2	1.5	269.3 ± 31.2	597.2 ± 68.4	160.84 ± 18.25	160.84 ± 18.25
2–3	2.5	267.3 ± 28.6	550.6 ± 59.8	147.18 ± 16.40	147.18 ± 16.40
3–4	3.5	143.6 ± 19.8	737.8 ± 82.5	105.95 ± 12.80	105.95 ± 12.80
4–5	4.5	331.7 ± 42.1	340.3 ± 38.6	112.88 ± 14.15	112.88 ± 14.15

5–8	6.5	163.4 ± 22.0	821.0 ± 95.2	134.15 ± 15.60	44.72 ± 5.20
8–12	10.0	508.9 ± 55.4	405.9 ± 46.5	206.59 ± 23.40	51.65 ± 5.85
12–18	15.0	339.6 ± 38.2	354.4 ± 41.2	120.37 ± 14.20	20.06 ± 2.37
18–24	21.0	227.6 ± 26.5	566.9 ± 62.4	129.05 ± 15.10	21.51 ± 2.52
24–36	30.0	1580.2 ± 145.0	76.2 ± 9.5	120.37 ± 14.80	10.03 ± 1.23
36–48	42.0	1422.8 ± 132.5	34.0 ± 4.8	48.37 ± 6.25	4.03 ± 0.52
48–72	60.0	2150.0 ± 180.2	Trace (<LOQ)	Trace	—

Data are represented as Mean ± SD across 5 healthy adult male volunteers.

Table S3. Mean cumulative urinary excretion percentage (%) and cumulative mass recovered over 72 h in human volunteers.

Time Point (h)	Cumulative NOR (mg)	Cumulative NOR (%)	Cumulative MET (mg)	Cumulative MET (%)
1	5.46 ± 0.82	1.36 ± 0.21%	0.071 ± 0.010	0.71 ± 0.10%
2	17.79 ± 2.35	4.45 ± 0.59%	0.232 ± 0.028	2.32 ± 0.28%
3	31.17 ± 3.80	7.79 ± 0.95%	0.379 ± 0.042	3.79 ± 0.42%
4	40.45 ± 4.90	10.11 ± 1.23%	0.485 ± 0.055	4.85 ± 0.55%
5	47.84 ± 5.65	11.96 ± 1.41%	0.598 ± 0.068	5.98 ± 0.68%
8	56.45 ± 6.70	14.11 ± 1.68%	0.732 ± 0.082	7.32 ± 0.82%
12	76.29 ± 8.95	19.07 ± 2.24%	0.938 ± 0.105	9.38 ± 1.05%
18	84.91 ± 10.12	21.23 ± 2.53%	1.059 ± 0.118	10.59 ± 1.18%
24	90.61 ± 11.20	22.65 ± 2.80%	1.188 ± 0.132	11.88 ± 1.32%
36	98.08 ± 13.40	24.52 ± 3.35%	1.308 ± 0.145	13.08 ± 1.45%
48	99.55 ± 14.80	24.89 ± 3.70%	1.357 ± 0.150	13.57 ± 1.50%
72	116.54 ± 18.51	24.89 ± 4.63%	1.740 ± 0.920	13.59 ± 9.27%

Data are represented as Mean ± SD across 5 healthy adult male volunteers over 72 h.

Table S4. Factor selection diagnostics: Core Consistency Diagnostic (CORCONDIA %) and percentage of explained variance (%Fit) as a function of the number of factors (F).

Factors (F)	XNOR: CORCONDIA (%)	XNOR: Fit (%)	XMET: CORCONDIA (%)	XMET: Fit (%)
1	100.0%	92.45%	100.0%	88.12%
2	98.7% (Selected)	99.92%	99.2%	96.40%
3	41.5% (Overfitted)	99.94%	94.2% (Selected)	99.85%
4	12.3%	99.95%	38.6% (Overfitted)	99.89%
5	0.0%	99.96%	8.4%	99.91%

The calibrated concentration ranges are 1500–6000 ng mL^{-1} for NOR and 20–80 ng mL^{-1} for MET. Samples below these boundaries are exploratory challenge samples, not extensions of the calibrated

range. Estimated LOQ values below the lowest calibrators do not independently establish validated lower limits of quantification.

Table S5. Calibration curve regression parameters for PARAFAC sample-mode loadings (a_if) vs. nominal drug concentrations in spiked human urine.

Analyte	Linear Range (ng mL⁻¹)	Slope (s_n ± SD)	Intercept (± SD)	Correlation (R^2)	RMSEC (ng mL⁻¹)
Norfloxacin (NOR)	1500–6000	0.00341 ± 0.00004	0.124 ± 0.015	0.9981	70.77
Metoclopramide (MET)	20–80	0.01285 ± 0.00009	0.048 ± 0.006	0.9995	0.86

Table S6. Parameter breakdown and scoring profiles for multi-metric sustainability and applicability tools.

Metric Tool	Score Obtained	Verbal Verdict	Key Drivers / Favorable Attributes
AGREE	0.68	Moderate–High Greenness	Zero organic solvent, 10-fold aqueous dilution, non-invasive biofluid, low energy fluorescence detection.
ComplexMoGAPI	69	Excellent Greenness	Total volume <2 mL aqueous buffer, zero flammable/toxic reagents, occupational hazard score minimal.
CaFRI	63.1	Average (Yellow)	Low electrical carbon emission (150 W Xenon lamp vs. high-power LC-MS/MS), no organic solvent manufacturing footprint.
BAGI	75.0	High Practical Applicability	Standard cuvette spectrofluorimeter, ~1 min measurement speed, direct measurement without SPE/chromatography columns.

Table S7. Freeze–thaw stability evaluation of Norfloxacin (NOR) and Metoclopramide (MET) in spiked human urine across 3 consecutive cycles (n = 5; exploratory below-range NOR results included).

The NOR Low QC concentration of 1200 ng mL⁻¹ lies below the revised calibration range of 1500–6000 ng mL⁻¹. Results at this concentration therefore represent an exploratory below-range stability assessment rather than validation of stability within the revised quantitative range.

Samples were spiked at Low QC and High QC levels, frozen at −20 °C for ≥24 h per cycle, thawed unassisted at room temperature (22 ± 2 °C), diluted 10-fold with pH 3.0 buffer, and quantified using the established dual-submatrix PARAFAC models. Acceptance criteria: mean %RE within ±15% and %RSD ≤ 15% (ICH M10 Section 3.2.8).

Analyte	Nominal Conc.	Cycle 1 Found ± SD [%RE, %RSD]	Cycle 2 Found ± SD [%RE, %RSD]	Cycle 3 Found ± SD [%RE, %RSD]	Assessment against stated

					criteria
NOR	Low QC (1200 ng mL⁻¹)	1218.4 ± 42.6 [+1.53%, 3.50%]	1205.1 ± 49.8 [+0.43%, 4.13%]	1184.7 ± 52.3 [−1.28%, 4.42%]	Within stated numerical criteria; exploratory below-range result
	High QC (5500 ng mL⁻¹)	5542.1 ± 112.5 [+0.77%, 2.03%]	5489.3 ± 134.1 [−0.19%, 2.44%]	5421.6 ± 158.2 [−1.43%, 2.92%]	Pass (Stable, |%RE| ≤ 1.43%)
MET	Low QC (20 ng mL⁻¹)	19.65 ± 0.81 [−1.75%, 4.12%]	19.42 ± 0.88 [−2.90%, 4.53%]	19.28 ± 0.93 [−3.60%, 4.82%]	Pass (Stable, |%RE| ≤ 3.60%)
	High QC (75 ng mL⁻¹)	76.24 ± 1.84 [+1.65%, 2.41%]	75.82 ± 2.05 [+1.09%, 2.70%]	74.65 ± 2.21 [−0.47%, 2.96%]	Pass (Stable, |%RE| ≤ 1.65%)

Table S8. Quantitative performance and chemometric diagnostic comparison between Proposed Dual-Submatrix PARAFAC, Traditional Global PARAFAC, and Second-Order Standard Addition Method (SOSAM) for NOR and MET in human urine.

Evaluated using external validation spiked urine samples and representative 72-hour human excretion samples. Global PARAFAC fits a single 3-way array across λex 260–310 nm, λem 380–500 nm; SOSAM utilizes 3 standard additions per unknown sample.

Performance Metric	Proposed Dual-Submatrix PARAFAC	Traditional Global PARAFAC	Second-Order Standard Addition (SOSAM)
Tensor Architecture	Two partitioned sub-arrays (XNOR & XMET)	Single unified tensor (16 × 121 × 26)	Individual tensor per unknown sample
Core Consistency (CORCONDIA)	98.7% (XNOR, F = 2), 94.2% (XMET, F = 3; Table S4)	54.3% (reduced core consistency)	N/A (Sample-by-sample decomposition)
NOR Recovery Range (%RE)	100.02–105.77% (|%RE| ≤ 5.77%)	94.12–112.45% (|%RE| ≤ 12.45%)	98.15–104.20% (|%RE| ≤ 4.20%)
MET Recovery Range (%RE)	97.82–106.76% (|%RE| ≤ 6.76%)	61.30–148.50% (|%RE| ≤ 48.5%, Unacceptable)	96.80–105.10% (|%RE| ≤ 5.10%)
MET RMSEP (ng mL⁻¹)	2.38 ng mL⁻¹	28.65 ng mL⁻¹ (Severe error)	2.40 ng mL⁻¹
Analysis Time / Sample	~1.0 min per sample	~1.0 min per sample	6.0–8.0 min per sample (4× scans)
Suitability for 72 h PK Profiles	Optimal (Single calibration set for 60 samples)	Fails due to variance-swamping of MET	240 scans for 60 samples (one unspiked scan plus three standard additions per sample)

Table S9. Assessment of matrix-associated response ratios (Matrix Factor, MF) across 6 individual human urine donor lots.

Matrix Factor (MF) = (sample-mode loading in spiked urine post-dilution) / (sample-mode loading in neat buffer at equivalent concentration). Interpretation of this ratio requires a common PARAFAC factor-scaling convention for matrix and buffer responses. Inter-lot variability in MF describes consistency across donors; it does not replace assessment of accuracy and precision within each donor lot at Low QC and High QC levels.

Donor Urine Lot	Specific Gravity	NOR Low QC (1200 ng mL⁻¹) MF	NOR High QC (5500 ng mL⁻¹) MF	MET Low QC (20 ng mL⁻¹) MF	MET High QC (75 ng mL⁻¹) MF
Donor 1	1.012	1.025	0.991	0.954	0.978
Donor 2	1.024	0.982	0.975	0.942	0.965
Donor 3	1.008	1.045	1.015	1.012	1.015
Donor 4	1.019	0.998	0.988	0.985	0.992

Donor 5	1.028	0.974	0.972	0.915	0.954
Donor 6	1.015	1.048	1.023	0.998	1.006
**Mean MF**	**—**	**1.012**	**0.994**	**0.968**	**0.985**
**SD**	**—**	**0.0328**	**0.0245**	**0.0384**	**0.0241**
**%CV**	**—**	**3.24%**	**2.46%**	**3.97%**	**2.45%**
**Descriptive inter-lot variability**	**—**	**CV ≤ 15%; below-range NOR**	**CV ≤ 15%; descriptive only**	**CV ≤ 15%; descriptive only**	**CV ≤ 15%; descriptive only**
